# This above All

**Published:** 1892-12-31

**Authors:** 


### The Hospital

AN INSTITUTIONAL JOURNAL OF

Science, Medicine, Nursing, and Philanthropy.

*For Hospitals, Asylums, Infirmaries, Dispensaries, Medical Practitioners, Students, Nurses, and the Charitable Public.*

VOL. XIII.—No. 327.] [DEC. 31, 1892.

### "THIS ABOVE ALL."

A MAN who stands quite close to the foot of a mountain can have no proper idea of its magnitude or its elevation. If he wishes to see its true proportions and sky-reaching heights he must stand a due distance away. In its closing days we can look back upon the greater part of the past year as from a distance, and can take count of its merits and demerits as they affect our country and as they affect ourselves. The year, many persons tell us, has been "bad." But "bad" in what way? Busihas been dull, and profits infinitesimal. This is a fact, or it is not a fact. Let us take it for granted that it is a fact. What then? Will the world come to an end because we have not made a great increase in our fortunes in a single twelvemonth?

Great Britain is a prosperous nation. She is prosperous because she is capable. Other nations are astonished at us, and we may well be astonished at ourselves when we contemplate our spreading arms on every continent, and our ruling wills in every land and sea. We have courage, we have the faculty for practical affairs, we can cultivate the ground, we can rule peoples, we can amass wealth everywhere, we can build up great political institutions, and we can defend them against all the world. It is not for people like us to hang the head and to belittle ourselves, because one year has happened to show depressing fortunes. Courage, undaunted courage; resolution, as of a hero in the grand struggle for victory, is the attitude of mind which befits those whom high Providence has so manifestly placed in the fore-front of the world's onward march.

It is sometimes said that the man who is truly great is not conscious of his greatness, and the same reasoning is extended to the nation. The reasoning is not sound; the fact is not true. If a man is truly great he does great things: and is he so stupid as not to know that they are great things? On the contrary, he chooses things for his doing because they are great, and because he is conscious of powers and a high calling. It is, it must be so with a nation. We scale the mountains of the world's difficulties, and establish ourselves on their heights because we feel at one and the same time the irresistible call to duties and the unquenchable capacity for deeds.

Can we be daunted then by the checks and difficulties of a single year? Is it possible for us to withold our hands from imperative good works because our coffers are not full to repletion? It is not the fullest coffers which produce the stoutest hearts. It is the sense of duty which puts the shoulder to the wheel in the stiff hill, and which keeps it there though the load threaten to crush out soul and life. The man with a conscience and a will knows no failure either of purpose or of effort. "His not to ask the reason why: his but to do or die," if need be.

The purpose we have before us at the present moment does not call us to "do and die," but to "do and live." We who, as a people, spread ourselves abroad like the floods of winter escaping from the beds of the rivers, must constantly see to it that our base of operations is strong and secure. England must have a great people at home if she is to continue to be great abroad. Only a healthy people can be a great people. The sick in body are bound to be sick in mind. The delicate man may be a genius. But granting that this is sometimes so by accident, yet even if it were so as a rule it is none the less true that the geniuses do not conquer the world. It is the workers, the steady, intelligent purposelike, day-by-day workers who sweep everything steadily and surely before them until they possess all the land.

Will it be conceded that the strongest defence we can have at our base of operations is a populous nation of healthy, happy, and duty-loving people? And will it be further conceded that the health of the people is only to be developed and conserved by intelligent understanding of the powers and limitations of the human body? And will it be conceded yet once more that a body of intelligent health-conservators, provided with means—of which hospitals are the chief—for suppressing illness promptly and guarding and strengthening health, is one of the very chiefest of all State resources in a vast and thickly crowded population? Let these things be granted—and who can deny them?—and we see at once that hospitals assume an importance which we never perceived before, and which demonstrates itself irresistibly like a vast mountain against the gleaming background of the sky.

"This above all, to thine own self be true." A stock quotation, but an inspired word! Englishmen must be addressed seriously, because they take themselves seriously. We always address them seriously in these pages, and we do it because we have the profoundest conceivable respect for the national character. Our fellow-countrymen are asked to be just and true to themselves on the hospital question. That question is a trust; it is a trust given into our hands by the noblest of the men of past generations. They planted that we might reap, but they hoped that we, too, should plant in our generation that those who follow us might also reap and plant. Our fathers planted well, with wise forethought. What could they have planted better than our voluntary and endowed hospitals? Have any other institutions of a national character ever served us better? We owe to these hospitals all our modern medical science; we owe to them much of the general science and culture of the times; above all, we owe to them the public and private health which distinguishes our own from all past ages, and which robs pestilence of its terrors and disease of much of its power to kill.

But we must be true also to our instinct of self-preservation; a great and imperative instinct; the vicegerent of Providence to preserve the race alive. That character is feebleness itself which has not a firm grip of life. We must live, or we cannot do the work which life apportions us. The prime necessary is life, and the second healthy life. This prime necessary hospitals exist to supply. They are the head and front and source of healthy physical and mental life. They constitute the material body and objective organism in which the modern health-spirit lives and works. When they languish that modern health-spirit is feeble; if they should die it would in all probability vanish to return no more.

The strongest of our arguments we retain for the last. Englishmen, Britons, must stand firmly by their hospitals, because conscience and duty lay this obligation upon them. Self-interest, says the cynic, is the most powerful of all motives to which appeal can be made. In the business of good works we affirm that self-interest is nowhere. The hospitals of this country are not maintained by self-interest. They are maintained by love, by conscience, by duty. Is there any reason to be ashamed of such motives? Was Darwin ashamed of them? Is Huxley ashamed of them? Will any man but a fool even pretend to laugh at them? We made our Christmas appeal last week; or, rather, we allowed the hospitals to make appeal for themselves. To-day, the last day of the year, we open wide the hospital doors once more, and ask all good men and women to look once more and see for themselves the work which is there being done, and which without their help must inevitably cease to be done. If Englishmen do not help we shall not blame them but ourselves for our feeble advocacy. They will help—they cannot do otherwise—if the truth can only be brought home to their minds. If anything could convince them it would be a sight of the work itself which is done in hospitals. Why, then, not go and see the work? There are 119 hospitals whose doors stand open to welcome you night and day. They have nothing to conceal; they have everything to gain by publicity. Go and see them; see their work; find out their needs; and, as Providence has blessed you with means, be true to your deepest and best convictions, and let duty and charity prevail.

